# Multilocular Sero-Cystic Ovarian Tumor Successfully Treated by Means of the Electropuncture

**Published:** 1874-09

**Authors:** Plym. S. Hayes

**Affiliations:** Chicago


ARTICLE II.—*Multilocular Sero-Cystic Ovarian Tumor successfully treated by Means of the Electropuncture.* By PLYM. S. HAYES, M.D., Chicago.

Miss S——, a medical practitioner, aged 40, consulted us Feb. 21st, 1874, with regard to an abdominal tumor. She is of nervo-sanguine temperament, the nervous predominating; a little below the medium height; of previous good health, the most severe illness she had ever experienced being an attack of cholera, seven years previous.

She commenced menstruating when thirteen or fourteen years old, and has menstruated regularly ever since without pain, until December 1st, 1873, at which time her attention was called to a tumor near the crest of the right ilium; since then, the menstrual periods have recurred at shorter intervals, and the discharge has been "dark and grumous for the first few hours, and quite profuse."

She has been troubled with a slight leucorrhœa for a number of years.

When the tumor was first discovered it was about the size of an orange, and the patient could distinguish through the abdominal walls a connection between it and the uterus.

About the middle of December, on jumping from a carriage she felt the tumor fall from its first position—at the crest of the right ilium—and in the course of two or three days it was detected in the left iliac fossa.

The tumor is at times the seat of a dull heavy pain, increased by pressure.

The patient says she can trace the outlines of three distinct lobes; these could be distinguished four weeks after the discovery of the tumor.

For the last two years she has experienced pain in the neighborhood of the rectum. She has lost considerable flesh since the discovery of the tumor.

She had diagnosed the growth as a multilocular sero-cystic ovarian tumor. Our first examination confirmed her diagnosis.

As her general health had suffered somewhat, she was advised to rest, and the electro-thermal bath was prescribed, during which the faradaic current was passed transversely through the tumor; after this the positive electrode, surrounded by a sponge, was placed on the abdomen over the growth, while the electrodes opposite the tumor were made negative; this treatment was preceded and followed by the use of the general currents. For about two weeks, under this treatment, the tumor decreased in size and the general health of the patient improved. At this time the menses appeared, and the tumor regained its former size.

In an examination prior to operating, Dr. A. Reeves Jackson was invited to examine the patient with us; which he did March 11th.

The abdomen was as large as that of a woman five months pregnant. The growth extended an inch and a half above the umbilicus when the patient was supine. As the abdominal walls were quite thin, the outlines of the tumor were easily distinguished. Three lobes could be distinctly outlined: one in the right iliac fossa, which had attached to its inferior portion a pedicle passing into the cavity of the pelvis; one to the left of the median line, connected by a band to the first named lobe: and a third—the original tumor—below this band and just above the pubis; this lobe was felt to be nodulated.

Fluctuation could be distinguished in the first two when palpation was employed over each separately, but not when the hand was placed over the one and the other percussed.

Digital examination revealed the fact that the os uteri was directed backward, pressed firmly against the rectum, and congested. Bimanual examination demonstrated that the tumor was not uterine. The sound was introduced into the uterus, and the tumor moved from side to side without producing any marked movement of the sound. The uterus was of normal depth.

The first operation took place at 5.30 P. M., March 13th. The patient was placed on her back on the table and her abdomen exposed. Ether spray was used to produce local anæsthesia over the points selected for the introduction of the needles.

Two needles were used, one insulated, and the other uninsulated, and attached to the same pole—negative—by means of a *serres-fines* conductor. The needles were introduced by Dr. Jackson. The insulated needle was introduced one inch and three-quarters into the abdominal walls and penetrated the right cyst, on a line connecting the anterior superior spinous processes of the ilia, and five inches from the right anterior superior spinous process. The uninsulated needle was introduced two inches, penetrating the left cyst in the same line with the other needle, three and a half inches from the left anterior superior spinous process.

Following the introduction of the needles the positive electrode, surrounded by a moist sponge, was placed over the right sacro-iliac articulation. The current from a battery of twenty-one Hill elements—with a galvanometer and rheostat in an accessory circuit, with a resistance of 2100 B. A. units and that of the galvanometer coil in this circuit—was applied for three minutes, when nine more elements were added, and the current from thirty elements continued for twelve minutes longer. The skin became whitened and raised around the uninsulated needle, while it remained depressed and unchanged around that which was insulated. A few drops of a clear serous fluid exuded on the withdrawal of the insulated needle. There was no hemorrhage from the needle punctures. Before the operation the pulse was 112 to the minute, during the operation 108, and afterwards 112.

For three nights and two days following the operation there was marked diuresis and diaphoresis. The pulse during this time was not less than 88, nor more than 112 to the minute. There was no pain after the operation, and for the first twenty-four hours afterward she suffered less than usual.

During the afternoon of March 14th she sat up in a chair, and received electrical treatment. Her feet were placed in warm water, into which the positive electrode had been placed, while the negative, enclosed in a sponge, was applied over various portions of the tumor for five minutes, with a galvanic current from twenty-one elements. Then the negative was placed in the foot bath and the positive held in the hands for ten minutes. This treatment was again given on the following afternoon.

When she first sat up, the day following the operation, she said that she felt a drawing sensation as though an adhesion were forming at the point where the insulated needle was introduced.

For some time prior to this treatment the patient had found it necessary to take a lunch between regular meals, probably on account of the rapid growth of the tumor; but since March 16th she has not felt the need of it.

March 21st, Saturday, she left the city on a visit, and returned

Monday. She said that she had not felt so well since she left, and that her abdomen was larger.

At 5.45 P. M., March 23d, an insulated needle was introduced seven-eighths of an inch into the abdominal walls—penetrating the right cyst of the tumor—about one-half of an inch from the former point of the introduction of the insulated needle. The current from thirty elements, with the same resistance in the accessory circuit, and applied as before, was continued for twelve minutes.

Before the operation the pulse was 92, after it, 112, and during the following evening, 96. This operation was followed, as in the previous case, by marked diuresis and diaphoresis, which continued for nearly two days.

At 4.15 P. M., April 9th, an insulated needle was introduced to the depth of one inch, penetrating the left cyst, half an inch nearer the median line than the point of introduction of the uninsulated needle. The current from thirty elements was employed, as in the former operations, and continued for fifteen minutes. The pulse before the operation was 118; after it, 100. No diuresis followed this operation; the patient, however, was in a gentle perspiration for some time afterward.

April 18th, at 4.30 P. M., the fourth and last operation was performed. An insulated needle was introduced to the depth of one inch and a quarter into the abdominal walls, one-half of an inch from the last puncture on the right side, and penetrating the right cyst. A current from thirty elements was employed as before for fifteen minutes. The pulse was constant at 98. The patient labored under more nervous excitement than at any previous operation. This was probably due to the proximity of her menstrual period. There was before the operation distinct fluctuation in the right lobe, while the left lobe could hardly be distinguished.

April 24th—She has just ceased to menstruate, and the tumor appears to be as large as before the last operation.

April 26th—The tumor has diminished much in size.

April 29th—Dr. Jackson made an examination through the abdominal walls. *The left cyst could not be found.* While using considerable force in trying to ascertain the size and position of the now greatly diminished right cyst it was ruptured with an audible sound. Following the rupture there was dullness on percussion at the most dependent portion of the abdomen, which was most marked when the patient was lying on her right side. The pulse was not accelerated, and there was no systemic disturbance following the rupture.

May 1st—The patient has voided more urine than usual, during the last two days. No tumor can be detected, and the ovary somewhat enlarged can now be felt. Percussion does not reveal any dullness above the pubis when in an erect position.

June 20th—An examination made this evening failed to discover any tumor. The right ovary could be distinguished through the abdominal walls, displaced to the left, and somewhat enlarged. No fluctuation could be felt, and the abdomen was resonant on percussion over the entire region of marked dullness prior to the last operation. The abdominal walls have become thickened and have lost the bluish cast which they had before the operations. Her menstrual periods have assumed their former regularity as regards time, and quantity, and quality of the discharge. Her general health is much improved, and during the last few weeks she has been gaining in flesh. During the time prior to, between and subsequent to the operations, the electro-thermal bath was used as an adjuvant to improve the general health and relieve the uterine and ovarian congestion.

July 21st—Two examinations have been made since the last notes were taken, at which time no cysts could be found. To-day Dr. Jackson made a thorough examination. He concurred in our opinion, that the cysts had been destroyed.

Four operations were performed. At the first, two needles were introduced, and subsequently only one neeedle was used at each operation. The longest period that intervened between any of the operations was seventeen days, which occurred between the second and third. Nine days intervened between the first and second and between the third and fourth operations. The needles used were insulated to within half an inch of their point, save in the first instance, when one of them was uninsulated. We used the insulated needles in preference to the uninsulated, because the punctures made by the former healed more readily than those made by the latter, on account of the uninsulated needle disorganizing the tissues in its immediate proximity. In all, two needles were introduced into the left, and three into the right cyst. At no time was there any peritoneal inflammation set up.

During the operations the most pain was felt at the place where the positive electrode, surrounded by a moistened sponge, was applied; this produced a burning sensation.

The needles were made negative rather than positive. First, because the negative always remains intact, while the positive, unless of gold or platinum, is disintegrated and therefore leaves some salt of the metal, which composes the needle, in the tissues—these salts are probably the oxides and chlorides. Second, hemorrhage is more apt to follow the removal of the positive than of the negative needle. Third, abscesses sometimes follow the introduction of the positive, but never that of the negative needle; and lastly, the irritation of the hydrogen liberated from the negative needle, if not the immediate cause of the absorption, continues the action commenced by the galvanic current.

We consider that the operation is attended with no more danger than is the introduction of the canula of an aspirator.

