# Erratum

**Published:** 1874-10

**Authors:** 


***Erratum.***

On page 517 (September number), in the caption of Article II., before "Plym. S. Hayes, M.D.," read *Justin Hayes*, *M.D.*, *and.*

